# *Drosophila mitoferrin *is essential for male fertility: evidence for a role of mitochondrial iron metabolism during spermatogenesis

**DOI:** 10.1186/1471-213X-10-68

**Published:** 2010-06-21

**Authors:** Christoph Metzendorf, Maria I Lind

**Affiliations:** 1 Comparative Physiology, Uppsala University, Norbyvägen 18A, 752 36 Uppsala, Sweden

## Abstract

**Background:**

Mammals and *Drosophila melanogaster *share some striking similarities in spermatogenesis. Mitochondria in spermatids undergo dramatic morphological changes and syncytial spermatids are stripped from their cytoplasm and then individually wrapped by single membranes in an individualization process. In mammalian and fruit fly testis, components of the mitochondrial iron metabolism are expressed, but so far their function during spermatogenesis is unknown. Here we investigate the role of *Drosophila *mitoferrin (dmfrn), which is a mitochondrial carrier protein with an established role in the mitochondrial iron metabolism, during spermatogenesis.

**Results:**

We found that P-element insertions into the 5'-untranslated region of the *dmfrn *gene cause recessive male sterility, which was rescued by a fluorescently tagged transgenic *dmfrn *genomic construct (*dmfrn^venus^*). Testes of mutant homozygous *dmfrn^SH115 ^*flies were either small with unorganized content or contained some partially elongated spermatids, or testes were of normal size but lacked mature sperm. Testis squashes indicated that spermatid elongation was defective and electron micrographs showed mitochondrial defects in elongated spermatids and indicated failed individualization. Using a *LacZ *reporter and the *dmfrn^venus ^*transgene, we found that dmfrn expression in testes was highest in spermatids, coinciding with the stages that showed defects in the mutants. Dmfrn-venus protein accumulated in mitochondrial derivatives of spermatids, where it remained until most of it was stripped off during individualization and disposed of in waste bags. Male sterility in flies with the hypomorph alleles *dmfrn^BG00456 ^*and *dmfrn^EY01302 ^*over the deletion *Df(3R)ED6277 *was increased by dietary iron chelation and suppressed by iron supplementation of the food, while male sterility of *dmfrn^SH115^/Df(3R)ED6277 *flies was not affected by food iron levels.

**Conclusions:**

In this work, we show that mutations in the *Drosophila *mitoferrin gene result in male sterility caused by developmental defects. From the sensitivity of the hypomorph mutants to low food iron levels we conclude that mitochondrial iron is essential for spermatogenesis. This is the first time that a link between the mitochondrial iron metabolism and spermatogenesis has been shown. Furthermore, due to the similar expression patterns of some mitochondrial iron metabolism genes in *Drosophila *and mammals, it is likely that our results are applicable for mammals as well.

## Background

Iron is an essential micronutrient for almost all organisms and is used as a co-factor for many enzymes involved in redox-reactions. Its reactivity with hydrogen-peroxide also bears the potential to promote the formation of reactive oxygen species via the Fenton reaction. Reactive oxygen species in turn result in, protein, lipid and DNA damage that can lead to cellular dysfunction and damage to organs. Consequently, free iron levels must be kept at a minimum, while enough iron must be provided to processes that depend on it (reviewed in [1,2]).

Mitochondria are the sites of iron-insertion into protoporphyrin IX [2] and iron-sulfur cluster (ISC) biosynthesis [3,4] within eukaryotic cells and are, therefore, the subcellular compartments with the highest requirement for iron. Transport of iron into mitochondria, is facilitated by the mitochondrial carrier proteins Mrs3p and Mrs4p (Mrs3/4p) [5,6] in yeast. *MRS3/4 *genes have been shown to genetically interact with *frataxin *[7] in the delivery of iron to heme [8] and ISC synthesis [9] in mitochondria. At least in yeast, another less effective mitochondrial iron transport mechanism seems to exist, as *MRS3/4 *mutants only manifest phenotypes at low iron conditions [10].

In vertebrates two paralog genes that are homologs to *MRS3/4 *exist. *Mitoferrin1*, is mainly expressed in erythropoietic tissues and the *frascati *mutations in the zebrafish result in hemoglobinization defects, anemia and lethality [11]. Both *mitoferrin1 *and *mitoferrin2 *can rescue yeast *MRS3/4 *double mutants, indicating a similar function. Only ectopic expression of *mitoferrin1 *can rescue *frascati *mutants [11] and it was recently shown that mitoferin1 protein, but not mitoferrin2, accumulates in erythropoietic cells at amounts that can meet the need of mitochondria for iron in these cells [12].

In a previous study we found that *Drosophila melanogaster *and other invertebrates (i.e., sea urchin, *Caenorhabditis elegans*, bee, wasp, mosquito and flour beetle) have only one *mitoferrin *gene, which is most likely a functional homolog of vertebrate *mitoferrin2 *as invertebrates lack erythropoiesis [13]. Study of *Drosophila mitoferrin *(*dmfrn*) in insect cell culture showed that its dysregulation affects cellular iron homeostasis through the iron-sulfur cluster synthesis pathway [13].

Mitochondria play an important role during spermatogenesis. For example, defects in caspase activation involving the spermatogenesis-specific cytochrome gene *cyt-c-d *[14] or defects in mitochondrial fusion processes, involving the *fuzzy onions *gene product [15], result in male sterility with defects during spermatogenesis. *Drosophila melanogaster *testes are 2 mm long terminally blind tubes. Spermatogenesis starts at their apical tip where stem-cell divisions give rise to germ cells [16]. Each germ cell is contained in a cyst of two somatic cyst cells [17] and undergoes four mitotic divisions, resulting in 64 syncytical spermatids after meiosis [18]. Mitochondrial fusion processes in spermatids result in two giant mitochondrial derivatives per spermatid that furl up to form the nebenkern. During elongation, the mitochondrial derivatives unfurl along the flagellar axoneme. As the individualization complex progresses along the length of the spermatids, each spermatid is wrapped in its own membrane, the minor mitochondrial derivative is depleted of most of its material, and other organelles and most of the cytoplasm are removed from spermatids and accumulate in the cystic bulge, which is cast off at the end of the spermatids as a waste bag [18]. After coiling, individualized spermatids are released from their cyst and stored as mature sperm in the seminal vesicle.

Here we report on a function of *dmfrn *during spermatogenesis and characterize its expression in different fly tissues as well as within testis. We show for the first time that the mitochondrial iron metabolism is essential during spermatogenesis.

## Results

### P-element insertion *P{lacW}dmfrn^SH115 ^*in the 5'-untranslated region of *dmfrn *results in male sterility

Previously we have identified *dmfrn *(CG4963), the only *Drosophila *homolog of yeast *Mrs3/4 *and vertebrate *mitoferrin2*, and studied its role in cellular iron homeostasis in cell culture [13]. Little is known about the role of *mitoferrin2 *in the whole organism.

**Figure 1:**
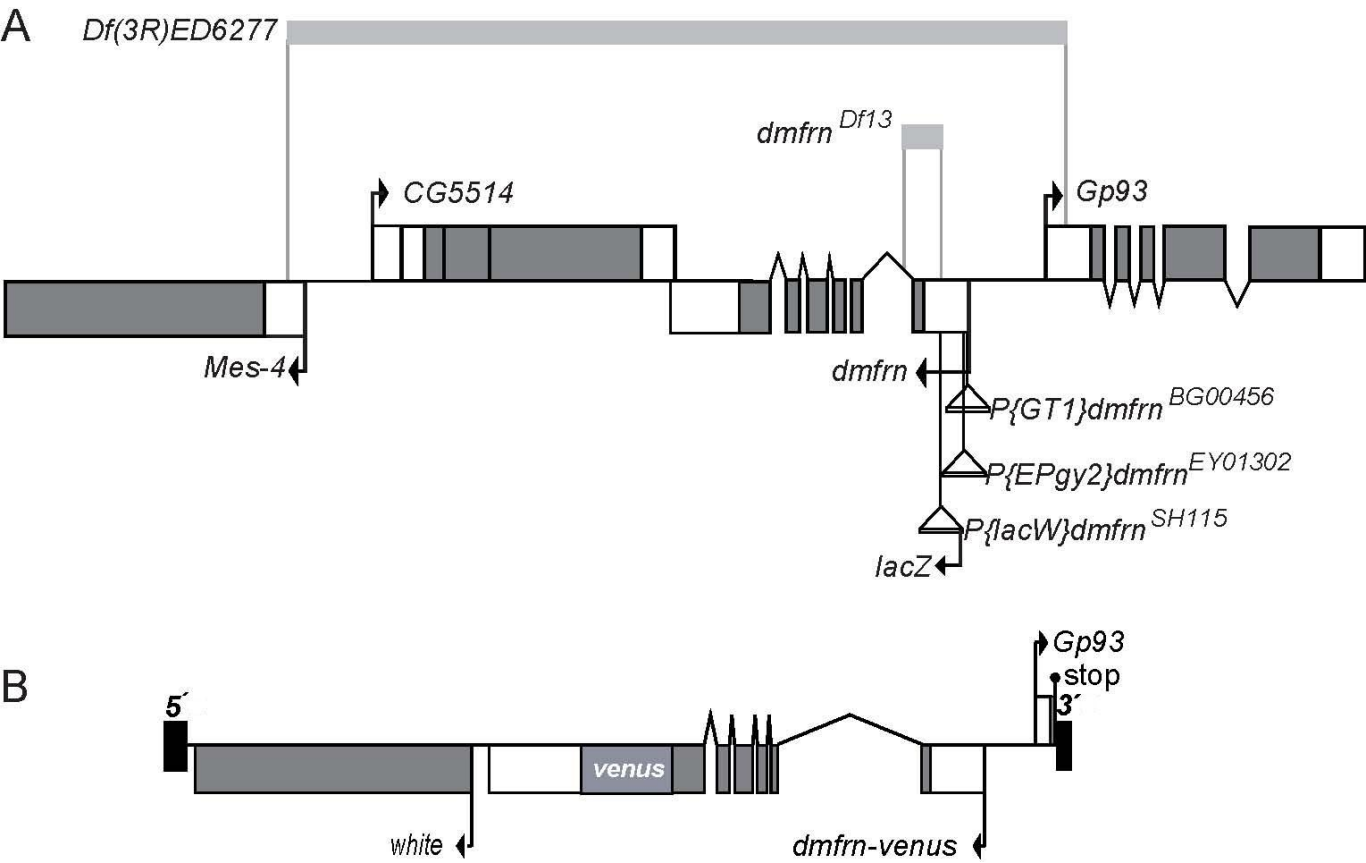
**The *Drosophila mitoferrin *(*dmfrn*) gene region and the genomic rescue construct *dmfrn^venus ^*A: P-element insertion sites and deletions in *dmfrn***. The gene *dmfrn *is encoded on the (-)strand and the coding sequence of *lacZ *in P-element *P{lacW}mfrn^SH115 ^*is encoded on the (-) strand as well (see Additional file 1 Figure A1A and A1D). **B**: The genomic rescue construct *dmfrn^venus ^*contains the gene region of dmfrn including the intergenic region between *dmfrn *and *Gp93*. The stop codon of dmfrn was substituted by the coding sequence of the fluorescent marker venus, resulting in the expression of a C-terminally venus-tagged dmfrn protein. The 5' and 3' inverted repeats of the P-element used for generation of transgene flies by P-element transposition are indicated by the black boxes.

To study *dmfrn*, we obtained four publicly available mutant alleles. Three are due to P-element insertions in the 5' untranslated region (5' UTR) of the *dmfrn *gene and one is a deficiency where *dmfrn *and a small part of the region in its proximity are deleted (*Df(3R)ED6277*; Figure 1A).

The insertion site of *P{lacW}l(3)SH115^SH115 ^*is downstream of the putative transcriptional start (about 252 bp;) but upstream of the start of the *dmfrn*-coding sequence (Figure 1A). It was recovered during a screen for recessive lethal genes [19], which would be in agreement with iron-sulfur clusters being essential co-factors [3] and the proposed general function of mitoferrin2 in mitochondrial iron transport in non-erythroid tissues [11]. P-elements *P{GT1}mfrn^B00456 ^*and *P{Epgy2}mfrn^EY01302 ^*, are both located closer to the putative transcriptional start of *dmfrn *(about 20 and 40 bp downstream, respectively, Figure 1A) and no phenotypes caused by the insertions themselves have been reported. *P{Epgy2}mfrn^EY01302 ^*contains an UAS sequence upstream of *dmfrn *and has been used in a gain of function screen, where overexpression of *dmfrn *in developing muscle apodemes caused muscle misdevelopment [20].

While cleaning the fly lines from background mutations by outcrossing them with wild type flies (strain *w^1118^*), lethality of *SH115^l(3)SH115 ^*flies was lost after only three generations. Therefore, it was unlikely that the reported lethality was caused by allele *SH115^l(3)SH115 ^*but by another unmarked mutation instead. Consequently, the allele *SH115^l(3)SH115 ^*should be referred to as *dmfrn^SH115^*.

To our surprise we failed to establish a homozygous stock of *dmfrn*^*SH115 *^flies, as male flies were recessive sterile and sterility was completely penetrant. Female *dmfrn*^*SH115 *^flies were fertile (100% fertile; n = 20), while male recessive sterility persisted even after outcrossing *dmfrn*^*SH115 *^flies for 13 generations to *w*^*1118 *^flies. To confirm that the allele *dmfrn*^*SH115 *^was indeed responsible for male sterility, a P-element excision screen, in which the P-element was re-mobilized, was carried out [21]. Independent lines of flies that had lost the marker for the P-element were established and analyzed by PCR for precise and imprecise excisions (i.e., P-element leftovers or genomic deletions) (Table 1). All precise excisions in *trans *to *dmfrn*^*SH115 *^rescued the recessive male sterility phenotype (Table 1). These results show that *P{lacW}dmfrn*^*SH115 *^causes male sterility.

**Table 1:** Transposon excision: test of fertility of white eyed flies (n.a. not analyzed).

Hop-out class	Total number of lines	Homozygous fertile	Hop-out/SH115 fertile
precise excision	11	10	11
transposon leftovers	7	1	1
deletion	3	n.a.*	n.a.

*the lines exhibited different degrees of lethality

We also analyzed fertility in male flies that carried *dmfrn*^*SH115 *^in *trans *to *Df(3R)ED6277 *or the small deletion *dmfrn*^*Df13 *^(Figure 1A), which was recovered during the hop-out assay, as well as flies that carried *dmfrn*^*Df13 *^in *trans *to *Df(3R)ED6277 *or were homozygous for *Df(3R)ED6277*. All of these combinations of *dmfrn *mutations resulted in male sterility (Table 2). To further support the role of *dmfrn *in male fertility and to rule out that *P{lacW}dmfrn*^*SH115 *^interferred with enhancer or repressor elements of other nearby genes, and thereby resulted in male sterility, we performed a genomic rescue experiment using a transgene fly line, which contains the gene region of *dmfrn *into which we had inserted a C-terminal venus tag (Figure 1B). This analysis showed that male sterility of homozygous *dmfrn*^*SH115 *^flies as well as sterility of *dmfrn*^*SH115*^/*dmfrn*^*Df13*^flies, *dmfrn*^*SH115*^/*Df(3R)ED6277 *flies, and *dmfrn*^*Df13*^/*Df(3R)ED6277 *flies was rescued by the transgene *dmfrn*^*venusB32 *^construct (Table 2). However, male sterility of homozygous *Df(3R)ED6277 *males was not rescued by *dmfrn*^*venusB32 *^(Table 2), indicating that the sterility of homozygous *Df(3R)ED6277 *males is likely caused by the deletion of one of the three other genes affected by the larger deletion. According to the FlyAtlas [22], the uncharacterized gene *CG5514 *is expressed in nerve tissue, ovaries and testes and is therefore the best candidate for male sterility of *dmfrn*^*venusB32*^*/+; Df(3R)ED6277 *flies. Inspection of testis squashes in these flies showed larger Nebenkerns associated with several normal sized nuclei (see Additional file 1 Figure A2), which is a sign for cytokinesis defects [23]. This indicates that *CG5514 *might be involved in cytokinesis during spermatogenesis.

**Table 2:** *dmfrn*^*venusB32 *^rescues male sterility of homozygous *dmfrn*^*SH115 *^as well as transheterozygote *dmfrn*^*SH115*^*/dmfrn*^*Df13*^, *dmfrn*^*SH115*^*/Df(R3)ED6277 *and *dmfrn*^*Df13*^*/Df(R3)ED6277 *male flies but not homozygous *Df(R3)ED6277 *male flies.

Genotype	% fertile flies
without rescue construct	
*SM1 Cy/+; dmfrn*^*SH115*^	0 (n = 37)
*SM1 Cy/+; dmfrn*^*SH115 *^*/dmfrn*^*Df13*^	0 (n = 50)
*SM1 Cy/+; dmfrn*^*SH115 *^*/Df(3R)ED6277*	0 (n = 30)
*SM1 Cy/+; dmfrn*^*Df13 *^*/Df(3R)ED6277*	0 (n = 10)
*SM1 Cy/+; Df(3R)ED6277*	0 (n = 17)
	
with rescue construct	
*dmfrn*^*venusB32*^*/+; dmfrn*^*SH115*^	97 (n = 33)
*dmfrn*^*venusB32*^*/+; dmfrn*^*SH115 *^*/dmfrn*^*Df13*^	100 (n = 25)
*dmfrn*^*venusB32*^*/+; dmfrn*^*SH115*^*/Df(3R)ED6277*	97 (n = 30)
*dmfrn*^*venusB32*^*/+; dmfrn*^*Df13 *^*/Df(3R)ED6277*	100 (n = 37)
*dmfrn*^*venusB32*^*/+; Df(3R)ED6277*	0 (n = 22)

**Figure 2:**
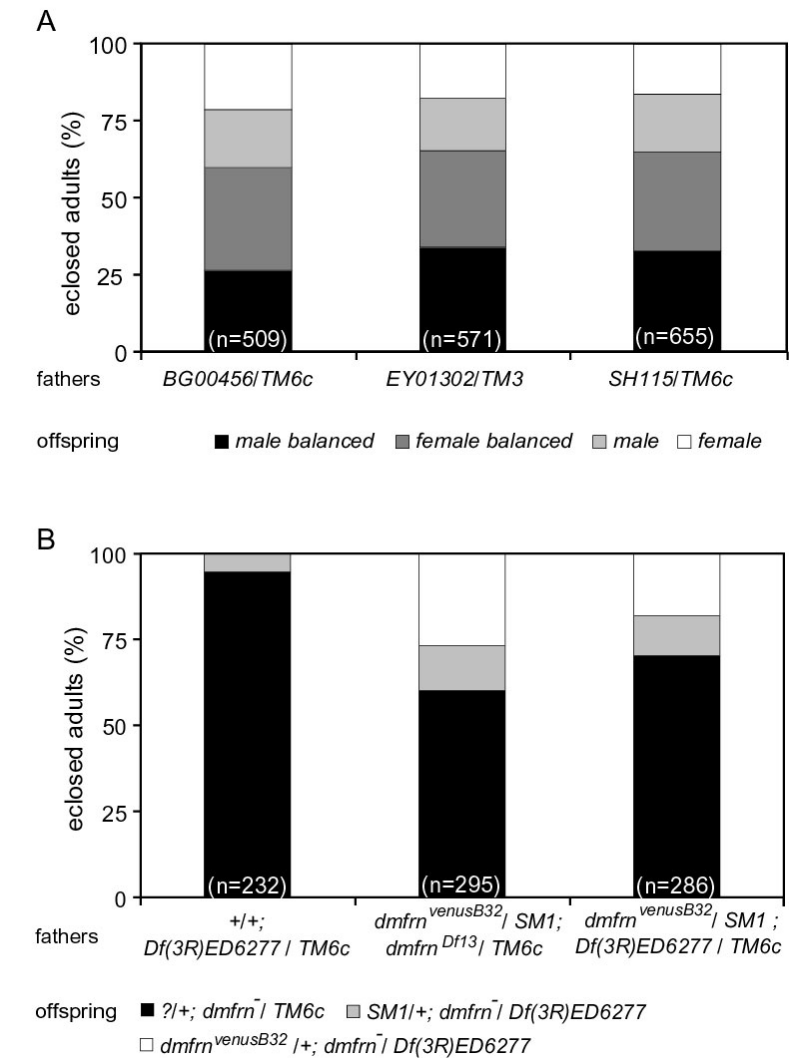
**Development of *dmfrn *mutants to adulthood**. Female *Df(3R)ED6277/TM6c *flies were crossed to male flies of the indicated genotype and allowed to lay eggs for three days. Numbers in parentheses show the total number of flies assayed per genotype. (**A**) Eclosed adult flies were collected and genotyped according to their genotypic markers. (**B**) Eclosed flies were sexed and genotyped according to their genotypic markers during an eclosion period of seven days. Male and female balanced: flies heterozygous for *dmfrn *mutations. Male and female *dmfrn^-^*: the indicated *dmfrn *mutation in *trans *to *Df(3R)ED6277*.

The fact that the emergence of homozygous *Df(3R)ED6277 *adult flies was lower than expected (6% instead of ~33%; Figure 2A), indicates that *Df(3R)ED6277 *causes lethality in addition to sterility. Flies with any of the three P-element insertions in *trans *to *Df(3R)ED6277 *did not show any obvious signs of lethality (Figure 2B). Male flies with the genomic rescue construct *dmfrn^venusB32 ^*on the second chromosome, and either the small or the large deletion on the third chromosome, were crossed to female *Df(3R)ED6277/TM6c *flies, and offspring were scored. The fractions of *dmfrn^Df13^/Df(3R)ED6277 *and homozygous *Df(3R)ED6277 *flies that carried the rescue construct (27% and 18%, respectively) were larger than the fractions of flies without the rescue construct (13% and 12%, respectively) (Figure 2A). These results show that the deletion of *dmfrn *results in partial lethality, indicating an essential role of *dmfrn *during development to adulthood.

As spermatogenesis uses many processes that are also needed during the normal development of an organism (i.e., cell proliferation, growth and morphogenesis) and the male sterility phenotype of *dmfrn*^*SH115 *^flies was completely penetrant, we continued investigating the role of dmfrn during spermatogenesis.

### *dmfrn*^*SH115 *^causes elongation defects during spermatogenesis

**Figure 3:**
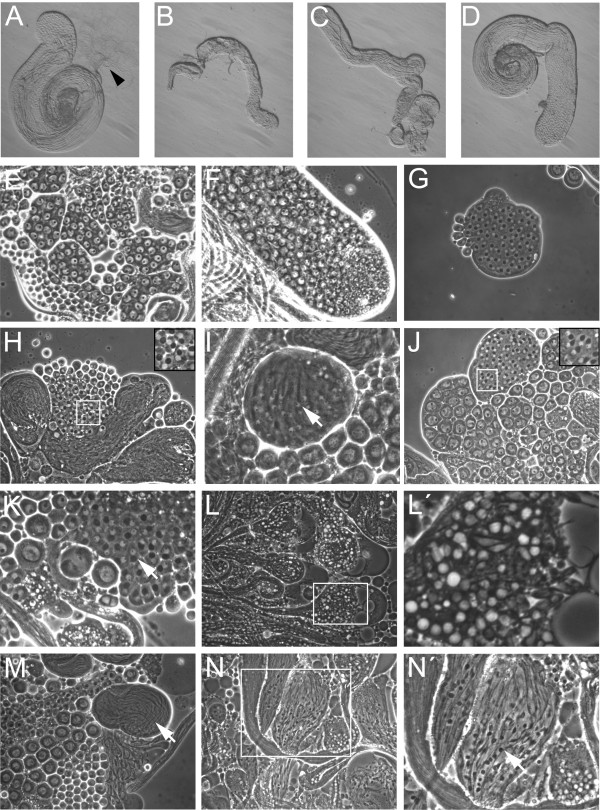
**Testes of *dmfrn*^*SH115 *^flies show defects during spermatogenesis**. Testis from a heterozygous *dmfrn*^*SH115*^*/TM6c *fly with mature motile sperm (**A **arrow head). Testes from homozygous *dmfrn*^*SH115 *^flies lack mature sperm, can be smaller (**B**) than WT, often have fewer elongated spermatids (**B **and **C**) or can look similar to WT testes (**D**). Phase contrast of testes squashes of *w*^*1118 *^(**E**, **G**, I and **M**) and *dmfrn*^*SH115 *^flies (**F**, **H**, **J**, **K**, **L**, **L'**, **N and N'**). Spermatocytes (**F**) and onion stage spermatids (**H**) of *dmfrn*^*SH115 *^tesis did not show any obvious defects and are indistinguishable from WT spermatocytes (**E) **and onion stage spermatids **(G)**. Early elongating spermatids of *dmfrn*^*SH115 *^testis show signs of delayed elongation (**J **and **K**; the arrow indicates the dark spot on the nucleous, which is characteristic for elongating spermatids, as can be seen in WT (**I)**). Spermatids of *dmfrn*^*SH115 *^testes frequently contained white spherical objects (**L **and **L'**). Membrane blebbing was observed on mitochondrial derivatives of elongating spermatids of *dmfrn*^*SH115 *^testes (**N **and **N' **arrow) but not on WT testes (**M, arrow**)

Male sterility can result from defects in mating behavior, abnormal anatomy of the sexual organs or defects during spermatogenesis [24]. Since homozygous *dmfrn*^*SH115 *^males were observed mating, behavioral abnormalities were ruled out as the cause of male sterility. Dissection of male flies revealed that the testes of homozygous *dmfrn*^*SH115 *^flies exhibited a defect during spermatogenesis of variable intensity resulting in the absence of mature sperm, whereas heterozygous testes looked normal and contained motile mature sperm (Figure 3A arrow head). The severity of the spermatogenesis defect in homozygous *dmfrn*^*SH115 *^flies ranged from testes that lacked properly elongated spermatids to those that looked almost like wild type (WT) testes but lacked mature sperm (Figure 3B to 3D shows some examples).

By analyzing testes squashes we found that *dmfrn^SH115 ^*spermatocytes (Figure 3F) and onion stage spermatids (Figure 3H) looked normal, whereas later stages of spermatids showed different defects. We found signs for delayed spermatid elongation (Figure 3J and 3K). Often, we observed abundant white structures of unknown origin in spermatid bundles (Figure 3L and 3L'). Sometimes mitochondrial derivatives of elongating and elongated spermatids had bulby protrusions (Figure 3N and 3N' arrow head) that are reminiscent of unelongated or improperly elongated mitochondrial derivatives. We also observed elongated spermatids that appeared normal, but never mature sperm. This is supported by the finding that all *dmfrn^SH115 ^*males were completely sterile. All of these results are indicative of an elongation defect in *dmfrn^SH115 ^*testes.

**Figure 4:**
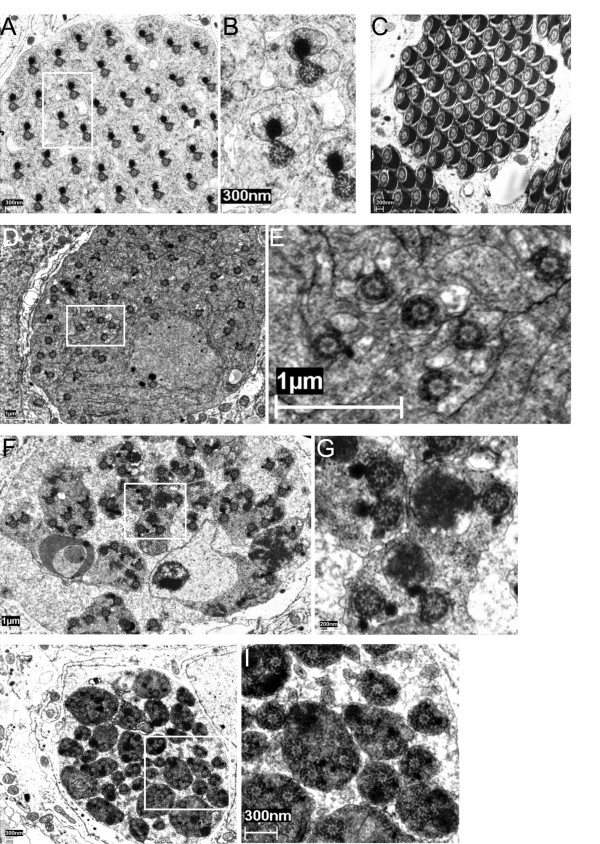
**Spermatids from *dmfrn*^*SH115 *^flies show defects in the maturation of the mitochondrial derivatives**. Transmission electron micrographs of ultra thin sections of testis from WT flies (**A-C**) and *dmfrn*^*SH115 *^flies (**D-I**). (**A**) Overview and (**B**) close-up of pre-individualization spermatids, showing the symmetric distribution of the spermatids within the cyst. The major mitochondrial derivative can easily be distinguished from the minor mitochondrial derivative by the accumulation of the electron dense stain. (**C**) Overview of individualized spermatids, which shows parachrystalline symmetry, a clearly visible major mitochondrial derivative and depletion of cytoplasm. (**D**) Overview and (**E**) close-up of *dmfrn*^*SH115 *^pre-individualization spermatids. No symmetry of the spermatids can be observed and accumulation of the paracrystalline structure to only a few major mitochondrial derivatives can be seen. (**F **and **H**) Overview and (**G **and **I**) close up of spermatids from *dmfrn*^*SH115 *^flies. Several axonemes are within the same spermatids and the accumulation of the paracrystalline structure within mitochondria is very heterogeneous.

At the ultrastructural level, elongated spermatids of *dmfrn*^*SH115 *^testes exhibited great morphological defects compared to those of WT testes. Spermatid cysts were unorganized (compare Figure 4A and 4C to Figure 4D and 4I). In testes of *dmfrn*^*SH115 *^flies, the major and minor mitochondrial derivatives, associated with axonemes were impossible to distinguish from one another in many cases. Often, one axoneme was associated with two mitochondrial derivatives of similar size, and both had accumulated the paracrystalline structure (Figure 4G), which is normally typical for the major mitochondrial derivative [25,18]. In some cases, one such mitochondrial derivative was extremely enlarged and contained heterogeneous accumulations of the paracrystalline structure (Figure 4G). Furthermore, in the same cyst the number of spermatids wrapped in the same membrane was not constant (Figure 4H), indicating that if individualization occurred, it was defective.

**Figure 5:**
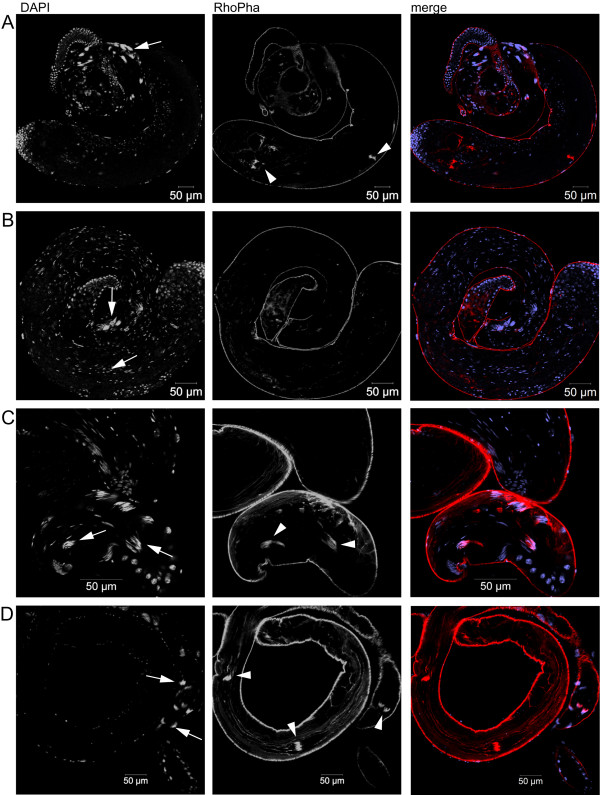
**Spermatid elongation defects in testis of *dmfrn*^*SH115 *^flies**. Nuclei were stained with DAPI (blue) and actin filaments were stained with rhodamine phalloidin (red). (**A**) Nuclei of elongated spermatids in WT testis are needle shaped and arranged in parallel (arrow). Individualization complexes (arrow heads) are visible as parallelz arranged cone-shaped structures. (**B**) Nuclei of elongated spermatids in testis of *dmfrn*^*SH115 *^flies are needle shaped but scattered (arrows). Individualization complexes were not observed. (**C**) Higher magnification of the base of a testis from *dmfrn*^*SH115 *^flies, showing the scattered, but needle shaped nuclei of elongated spermatids. Two nuclei bundles are associated with f-actin structures (arrows), which might be newly forming individualization complexes. (**D**) Parallel, needle shaped nuclei (arrows) and individualization complexes (arrow heads) are observed in *dmfrn*^*venusB32*^*/+; dmfrn*^*SH115 *^flies. Images were acquired by confocal laser scanning microscopy and represent optical slices through testes.

Defects during spermatogenesis can either be primary defects, which are a direct consequence of a mutation or they can be secondary defects, which are the result of primary defects [23]. To find out whether possible defects during individualization, as indicated by the above TEM results, were preceded by earlier defects that are independent of the elongation defects that we observed under phase contrast, we analyzed whole mount testis of *dmfrn*^*SH115 *^flies by confocal laser scanning microscopy. Nuclei and f-actin were stained with DAPI and rhodamin phalloidin, respectively. In WT testes, nuclei of elongated spermatids were needle-shaped and located at the base of the testis in parallely packed bundles (Figure 5A, DAPI) and individualization complexes present at different positions of elongated spermatids were apparent from well organized actin-cones (Figure 5A, RhoPha). Even though nuclei of spermatids from *dmfrn*^*SH115 *^testes were needle-shaped, they were often scattered over large areas of the length of the testes (Figure 5B, DAPI) or formed fuzzy bundles (Figure 5C, DAPI). Despite the presence of parallel organized nuclei in some mutant spermatid bundles, we did not observe actin-cones in the mutant testes we analyzed (Figure 5B and 5C, DAPI). However, in one testis we observed the associaton of f-actin with nuclei, which might be indicative for the formation of an individualization complex (Figure 5C). So, even though we could not see any actin cones, it may not be ruled out that some spermatids did form individualization complexes and underwent partial individualization. All defects observed in *dmfrn*^*SH115 *^testes were rescued in testes of *dmfrn*^*venusB32*^*/+; dmfrn*^*SH115 *^flies (Figure 5D).

A parallel organization of the nuclei at the start of individualization is required for the successful assembly of functional individualization complexes. From the above results, it is therefore likely that the defects observed by TEM in *dmfrn*^*SH115 *^testes are the result of the elongation defects.

### *dmfrn *is ubiquitously expressed with slightly higher expression in testes

Mitochondrial ferritin, an iron storage protein, is most abundantly expressed in testes of mice [26,27] and flies [28], indicating that the mitochondrial iron metabolism, in particular, might play a role during spermatogenesis in insects and mammals.

According to the FlyAtlas [22], *dmfrn *expression is lowest in testes, which was rather unexpected in light of our findings. This prompted us to verify *dmfrn *expression in different tissues by RT-RT PCR. We isolated RNA from heads, thoraxes, guts, malpighian tubules and testes of 2-3 days old virgin male flies and analyzed the expression levels of *dmfrn *and other iron metabolism related genes (i.e., *frataxin homolog *(*fh*), *Fer1HCH*, *Fer2LCH *and *Fer3HCH*) as well as the house keeping gene *RP49*.

**Figure 6:**
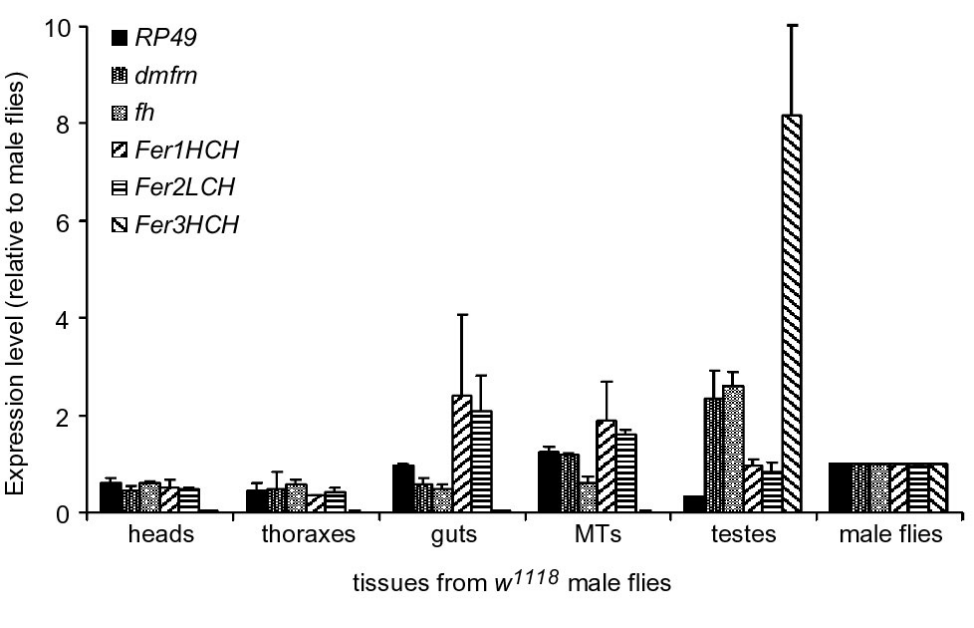
**Expression pattern of iron metabolism related genes in tissues of adult males**. cDNA was prepared from RNA extracted from dissected tissues of 2-3 days old adult male *w*^*1118 *^flies. MT = malpighian tubules. Gene expression in the tissues is relative to that of gene expression in whole flies. The data represent the mean of two independent experiments.

Mitochondrial ferritin (*Fer3HCH*) was expressed at extremely low levels in all tissues except for testes where its message was about eight times more abundant than in whole fly homogenates (Figure 6). This expression pattern is in agreement with that of a previous report [28] and is similar to the FlyAtlas expression data (about 10 times more transcript in testes, compared to whole flies of both sexes).

Transcripts of *Fer1HCH *and *Fer2LCH *were enriched about twofold in the gut and malpighian tubules but not in testes (Figure 6), which again is similar to the pattern reported in the FlyAtlas. Ferritins of *Drosophila *are involved in iron storage and iron transport (for a review on insect iron metabolism see [29]), and accumulate in the iron region of the midgut of iron loaded flies [30]. Higher expression of ferritins in the gut could therefore either be an indication for the mobilization of food iron to other tissues or iron storage.

The expression patterns of *dmfrn *and *fh *in our analysis (Figure 6) diverged from the FlyAtlas data. In all tissues, except for testes, both were expressed at levels similar to that of whole flies. In testes, message abundance of *dmfrn *and *fh *were about two-fold higher (Figure 6).

### *dmfrn *is expressed at increased levels in spermatids

The above mentioned results indicate that *dmfrn *expression in testes is higher than in other tissues. Testes contain both somatic cells; i.e, cyst cells and cells of the testis sheath, as well as germline cells of different developmental stages. Mitochondrial aggregation and formation of the giant mitochondrial derivatives take place in spermatids [24] and since we observed spermatid elongation defects, we suspected that mitoferrin expression could occur before or during this stage.

**Figure 7:**
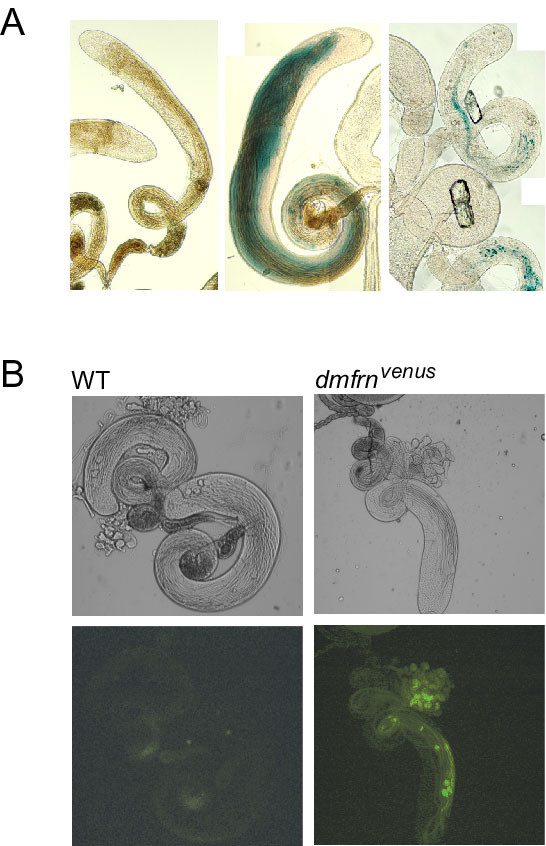
***dmfrn *expression in testes**. (**A**) X-Gal stained testis of *w*^*1118 *^(left) flies, heterozygous (middle) and homozygous (right) *dmfrn*^*SH115 *^flies. (**B**) Light microscopy (upper row) and fluorescent microscopy (lower row) of testes from flies that do not express *dmfrn*^*venus *^(WT) and flies that express *dmfrn*^*venus *^(*dmfrn*^*venus*^).

*P{lacW}dmfrn*^*SH115 *^contains the coding sequence (CDS) of the β-galactosidase gene (*lacZ*) [31] on the same strand as *dmfrn *(Figure 1A and see Additional file 1 Figure A1) allowing the visualization of the expression pattern of *dmfrn *in testes through X-gal staining. Blue stain was not detectable in testis of *w*^*1118 *^flies, which were used as a negative control (Figure 7A left). In testis from heterozygous *dmfrn*^*SH115 *^flies, blue stain accumulated in spermatids and was absent from spermatocytes and the rest of the genitalia (Figure 7A middle). X-gal staining of small testis from homozygous *dmfrn*^*SH115 *^flies showed the same pattern and only a few spermatids were elongated to a small degree (Figure 7A right).

To verify the expression pattern of *dmfrn *and to be able to study the localization of dmfrn protein during spermatogenesis in further detail, we examined testes from *dmfrn*^*venusB32 *^flies under the fluorescence microscope. First, we made sure that the signal of dmfrn-venus protein was clearly discernible from autofluorescence of testes by using *w*^*1118 *^flies as a negative control (Figure 7B left). A clear signal of dmfrn-venus protein was visible in elongated spermatids (Figure 7B right). This expression pattern is similar to the expression pattern of *dmfrn *obtained through the expression of *lacZ*, confirming that *dmfrn *is expressed late during spermatogenesis. Using confocal microscopy with increased gain, we were also able to detect dmfrn-venus protein in spermatocytes and the testis sheath (see Additional file 1 Figure 3).

**Figure 8:**
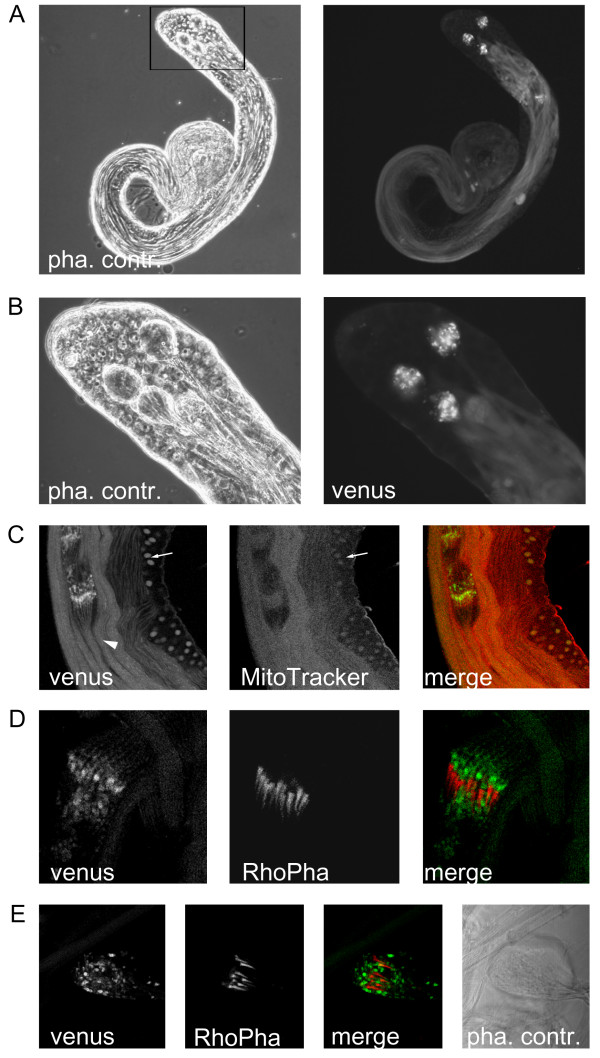
**Localization of dmfrn-venus protein in testis using conventional microscopy (A and B) and confocal laser scanning microscopy (C-E)**. (**A**) Phase contrast (pha. contr.) and fluorescence (venus, green) microscopy of a testis from a *dmfrn*^*venusB32 *^fly. dmfrn-venus protein accumulates in elongated spermatids and waste bags. (**B**) Higher magnification of the area indicated in (**A**), showing the accumulation of dmfrn-venus in waste bags. (**C-E**) Confocal laser scanning microscope images of *dmfrn*^*venusB32 *^expression in spermatids. (**C**) Expression of *dmfrn*^*venusB32 *^(venus, green) in whole mount testis. Mitochondria stained with MitoTracker Deep Red 633 (MitoTracker, red). *Arrow*: onion stage spermatids; *arrow head*: elongated spermatids. (**D**) Localization of dmfrn-venus inside the cystic bulge at the individualization complex. dmfrn-venus (venus, green) accumulates in mitochondrial whorls in front of the actin cones (RhoPha, red) of the individualization complex. (**E**) After individualization, dmfrn-venus (venus, green) accumulates in waste bags.

At higher magnification, we observed that dmfrn-venus protein accumulated in elongated spermatids, the region of spermatid individualization, and that a large fraction was disposed of in waste bags (Figure 8A and 8B). Closer inspection of whole mount *dmfrn*^*venusB32 *^testes, using confocal microscopy, confirmed these observations (Figure 8C to 8E) and showed that dmfrn-venus protein abundance was increased in nebenkerns of onion stage spermatids and in elongated spermatids (Figure 8C). During spermatid individualization, dmfrn-venus accumulated in mitochondrial whorls in front of the actin cones of individualization complexes (Figure 8D). At the end of spermatid individualization, the bulk of dmfrn-venus had accumulated in cystic bulges and ended up in waste bags (Figure 8E).

### Male fertility of hypomorph *dmfrn *mutants depends on food iron levels

Yeast *MRS3/4 *double deletions only cause a growth defect on low iron medium [10]. We were therefore interested to see whether food iron levels had any effect on fertility of the *mfrn^SH115 ^*mutant. We also wanted to test if the fertility of flies with the two other P-element insertions, which could be kept as homozygous stocks on normal food, were influenced by iron availability.

To be able to quantify and compare fertility of the different P-element mutants, we wanted to use a genetic background that was as similar as possible. Therefore, we crossed male *dmfrn^SH115^/TM6c *or *dmfrn^EY01302^/TM3 *or *dmrfn^BG00456^/TM6c *flies with female *Df(3R)ED6277/TM6c *flies. Eggs were laid on low iron food (food containing the iron chelator BPS), normal food and high iron food (~2.5 mM Fe^3+ ^as ferric amonium citrate (FAC)) to allow development of offspring under these different iron conditions. Males that carried the respective mutant allele over *Df(3R)ED6277 *were collected and fertility was quantified.

**Figure 9:**
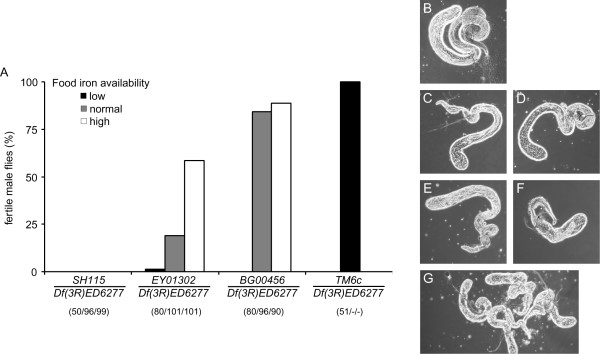
**Male fertility of hypomorph P-element mutants depends on food iron levels**. Female Df(3R)ED6277/TM6c flies were crossed to male flies of the genotypes *dmfrn*^*SH115*^*/TM6c*, *dmfrn*^*EY01302*^*/TM3 *and *dmfrn*^*BG00456*^*/TM6c*. Eggs were laid on normal potato fly food (N), on low iron potato fly food (140 μM BPS) or on high iron potato fly food (~2.5 mM Fe^3+ ^as FAC) for three days. (**A**) Adult male offspring of the indicated genotype were mated to *w*^*1118 *^virgin females (one male was crossed to two females) to quantify male fertility. Numbers in parentheses show the total number of single crosses per genotype. (B-G) Testes of flies reared on low iron food as observed under phase contrast (all images are of the same scale). Testes of *TM6c/Df(3R)ED6277 *flies (B), *dmfrn^BG00456^/Df(3R)ED6277 *flies (C and D), *dmfrn^EY01302^/Df(3R)ED6277 *flies (E and F) and *dmfrn^SH115^/Df(3R)ED6277 *flies (G) are small and contain, if any, few elongated spermatids.

*Df(3R)ED6277/TM6c *males raised on low iron food were completely fertile (Figure 9A), showing that iron starvation by itself did not cause male sterility.

Male sterility on normal food was low in *dmfrn^BG00456^/Df(3R)ED6277 *flies, higher in *dmfrn^EY01302^/Df(3R)ED6277 *flies and complete in *dmrfn^SH115^/Df(3R)ED6277 *flies (Figure 9A). Supplementation of food with iron increased fertility of *dmfrn^EY01302^/Df(3R)ED6277 *flies strongly, while low iron conditions reduced fertility of *dmfrn^BG00456^/Df(3R)ED6277 *and *dmfrn^EY01302^/Df(3R)ED6277 *males drastically (Figure 9A). *dmfrn^SH115^/Df(3R)ED6277 *male flies were completely sterile, regardless of the food they were raised on (Figure 9A).

We also observed testes of the different P-element mutants raised on low iron food. Testes of TM6c/Df(3R)ED6277 flies, which were used as a control, looked like normal WT testes with abundant elongated spermatids and mature motile sperm in the seminal vesicle (Figure 9B), showing again, that low iron levels alone do not cause defects during spermatogenesis. Testes of *dmfrn^BG00456^/Df(3R)ED6277 *and *dmfrn^EY01302^/Df(3R)ED6277 *flies contained very few elongated spermatids (Figure 9C and 9D and Figure 9E and 9F, respectively), whereas *mfrn^SH115^/Df(3R)ED6277 *testes were very small and did not contain elongated spermatids (Figure 9G). This, in turn, shows again that *dmfrn^SH115^*, which is further downstream in the 5' UTR of *dmfrn*, causes the strongest phenotype.

From these results, and the well established role of mitoferrins in the mitochondrial iron metabolism, we conclude that *dmfrn *and the mitochondrial iron metabolism are essential for spermatogenesis.

## Discussion

The yeast homologs of *dmfrn *and *frataxin homolog *(*fh*) have been shown to genetically interact in yeast during iron-sulfur cluster (ISC) biosynthesis [9] and heme biosynthesis [8]. Previously, we have shown that *dmfrn *rescues yeast *MRS3/4 *double mutants and that its overexpression alters cellular iron homeostasis [13]. In the current study we show that the mitochondrial iron metabolism plays a role during spermatogenesis for the first time directly, through the male sterility phenotypes caused by P-element insertions into *dmfrn *and the dependence of the hypomorph mutant on dietary iron, and indirectly, through the expression of *fh *and *dmfrn *in testes. This is even further supported by the high expression level of mitochondrial ferritin in testis (our results and [28]). The fact that *frataxin*, *mitochondrial ferritin *and *dmfrn*/*mitoferrin2 *are all expressed at higher levels in testes of *Drosophila *and mammals [32,26,27,11,33], indicates that the involvement of the mitochondrial iron metabolism in spermatogenesis is very likely conserved from insects to mammals.

Early studies of mammalian spermatogenesis indicated a nutritional function of iron during spermatogenesis. In human seminal plasma, levels of transferrin, an ubiquitous iron transport protein in mammals, are correlated with sperm abundance [34]. Furthermore, transferrin is produced in Sertoli cells ("nurse cells") of mammalian testes and delivers iron to germinal cells [35-37]. Defects in the mitochondrial iron metabolism, especially the iron-sulfur cluster synthesis pathway, result in cellular iron accumulation [38-40]. Increased iron uptake and accumulation in yeast *MRS3/4 *mutants have been shown [41] and might occur in *dmfrn *mutants. It has been shown that injection of iron into testes of rats results in sterility and tissue degeneration [42], and similar experiments with other metals do suggest that this might be a general effect of heavy metals [43]. However, the fact that nutritional iron loading of *dmfrn *mutant flies rescued the weaker male sterility phenotypes and that iron starvation enhanced these phenotypes, indicates that the spermatogenesis defects in the testis of *dmfrn *mutants is not the result of cellular iron overload. We interpret our results as further support for a nutritional function of iron during spermatogenesis. This would also be in agreement with the large variety within the testis development we observed (ranging from very small to WT-like testis that lack mature sperm).

The growth defect of yeast *MRS3/4 *double deletions develops only under iron limiting conditions, and it has been reasoned that other, still unidentified transporters with lower iron affinity could compensate for the lack in Mrs3/4p under iron replete conditions [10,6]. Deletion of *dmfrn *results in partial lethality, whereas flies with the P-element insertions in the 5' UTR of *dmfrn *are viable. Therefore, we suggest that there is residual *dmfrn *expression in the P-element insertion lines. In those lines with P-elements integrated closest to the putative transcriptional start site of *dmfrn*, expression might be high enough to allow nutritional iron loading to compensate for lower *dmfrn *expression, while *dmfrn *expression in *dmfrn^SH115 ^*testes would be insufficient to sustain spermatogenesis, even under iron loading conditions. Testes are heterogeneous microenvironments and the germ cells are contained within a pair of cyst cells during all stages of development, and the function of cyst cells in *Drosophila *is poorly understood. Therefore, it could very likely be that access of germ cells to iron is controlled by cyst cells, and that even iron loading of *dmfrn^SH115 ^*flies cannot provide enough iron for a low affinity transporter to compensate for the lack of dmfrn in *dmfrn^SH115 ^*testes.

Several properties and phenomena of spermatogenesis are likely to rely on mitochondrial iron metabolism: (i) Mitochondria of spermatids aggregate to form the giant nebenkerns through fusion processes that depend on mitochondrial activity [44], which in turn depends on the activity of the respiratory chain. Several complexes of the respiratory chain contain heme or ISC or both as prosthetic groups, linking energy metabolism directly with the mitochondrial iron metabolism. Insufficient *dmfrn *expression could lead to defects in the energy metabolism of the giant mitochondria and interfere with mitochondrial dynamics. (ii) Spermatids undergo dramatic morphological changes as they elongate to a length of almost 2 mm. This process is most likely very energy craving and, therefore, well functioning electron transport chains should be essential. If the energy metabolism is corrupted, elongation is likely to stop or slow down. (iii) Spermatids undergo an apoptosis-like processes during their individualization. The testis specific variant of the heme protein cytochrome c, encoded by the gene *cyt-c-d*, has been shown to be essential for spermatid individualization [45,14] and its function might be sensitive to heme deficiency. Even though we observed individualization defects that could hint to defects in the apoptosis-like process in spermatids of *dmfrn *mutants, it cannot be ruled out that preceding defects during spermatid elongation are the underlying cause.

The exact functions of the major and minor mitochondrial derivatives of insect sperm are not clear and several different hypotheses exist. Mitochondrial derivatives may be extremely efficient mitochondria or are degenerated mitochondria or modulate the undulation of the sperm tail in a species specific manner [25]. During spermatid individualization, a large part of the minor mitochondrial derivative is removed from spermatid tails and is disposed of in waste bags [18] and we found that a large fraction of dmfrn follows this portion of mitochondria. As the sperm tail is stripped from all of its organelles, except for the remaining part of the mitochondrial derivatives, mitochondrial transport is very likely to become obsolete. On the other hand, remaining mitochondrial carriers could clean the cytoplasm from left-over substrates. In *Drosophila*, ferritin resides in the endoplasmatic reticulum (ER) and can be secreted [46]. Using testes of *Fer1HCH^G188 ^*flies that express GFP-tagged Ferritin Heavy Chain Homolog protein [30], we were able to identify ferritin in close proximity to mitochondrial whorls and its accumulation in waste bags (see Additional file 1 Figure A4). The close proximity of the ER to mitochondrial whorls could be an indication of iron transfer from the Fer1HCH/FerLCH pool to mitochondria to maintain a functional respiratory chain and active cytochrome-c-d.

A recently published article reports that the copper transporter Ctr1C in *Drosophila*, is essential for male fertility in a *Ctr1B *mutant background [47]. Furthermore, Ctr1C locates to the cytoplasmic membrane and is expressed in spermatids and elongating spermatids [47], indicating that metals, in general, play an important role during spermatogenesis.

## Conclusions

From our findings we conclude, that *Drosophila *mitoferrin and the mitochondrial iron metabolism are essential during spermatogenesis. *Drosophila *and mammalian spermatogenesis have several processes in common [48] and genes involved in the mitochondrial iron metabolism are expressed in testis of both vertebrates and *Drosophila*. Therefore, it is not unlikely that our findings are applicable for vertebrates as well. Our study provides a first insight and tools in the form of characterized fly mutants, that will aid further investigations concerning the role of iron, and specifically mitochondrial iron metabolism during spermatogenesis.

## Methods

### Fly strains

*w^1118 ^*and *w^1118^; Vno/TM6c Sb, Tb *flies were obtained from M.S. Dushay (Illinois Institute of Technology) and *w^1118^; wg/CyO; Δ2-3 Sb/TM6c, Tb *flies and *w^1118^*; *Sco*/*SM1*, *Cy*; *Vno*/*TM3*, *Sb *flies were obtained from P. Kylsten (Södertörns Högskola, Sweden). *l(3)SH115 *and *Df(3R)ED6277 *flies were obtained from Szeged *Drosophila *stock center, Hungary. Stocks of *dmfrn^EY01302 ^*and *mfrn^BG00456 ^*flies were obtained from the Bloomington *Drosophila *stock center, USA. *w^1118^*; *Sco*/*SM1*, *Cy*; *Vno*/*TM6c*, *Sb Tb *were made by conventional fly genetics. We genotyped *dmfrn^SH115^*, (*SH115^l(3)SH115^*) *dmfrn^EY01302 ^*and *mfrn^BG00456 ^*flies to confirm the stocks (see Additional file 1, additional methods for genomic DNA extraction and PCR protocol).

The ~11 kbp large deletion *Df(3R)ED6277 *at cytogenic map position 98B6 was generated by recombination as part of the DrosDel project [49,50]. It removed genes *dmfrn *and *CG5514 *completely and parts of the putative 5' UTRs of *Mes-4 *and *Gp93*. Because *Df(3R)ED6277 *was uncharacterized and unverified, we performed PCR confirmation (see Additional file 1, additional Figure A1B and A1F) as proposed by Ryder *et al*. [50] and sequenced the products, confirming the integrity of the recombination product.

Flies were kept on standard potato sucrose medium in a 12 h/12-h light/dark cycle. Fly stocks were kept at 18°C. Experiments and crosses were carried out at room temperature (22-25°C).

For histostaining and other microscopic work, adult male flies were separated from female flies after eclosion and testes were removed from one to three days old flies in PBS buffer using sharp tweezers.

### P-element reversion screen

P-element reversion [21] was carried out by crossing female *w^1118^; dmfrn^SH115 ^*virgins with *w^1118^; wg/CyO; Δ2-3 Sb/TM6c Tb *male flies that carry the immobilized transposase source Δ2-3 [51]. F1 males of the genotype *w^1118^; CyO/+; Δ2-3 Sb/dmfrn^SH115 ^*(mosaic expression of white) were crossed to virgin *w^1118^; Vno/TM6c Sb, Tb *female flies. Single white eyed male F2 of the genotype *w^1118^; +/+; ?/Vno *were crossed to virgin *w^1118^; Vno/TM6c Sb, Tb*. Finally, F3 *w^1118^; ?/TM6c Sb, Tb *siblings were used to establish lines. These were then analyzed by PCR for the absence of *P{lacW}SH115^l(3)SH115^*.

### Fertility assay

Single 3-4 days old male or female flies, collected from flies reared on indicated food sources, were mated with 2-3 virgin *w^1118 ^*female or 2 male *w^1118 ^*flies reared on normal food, respectively. After 5-7 days of mating, the fraction of fertile flies was determined by the presence of larvae.

### Transgenic *dmfrn^venus ^*flies

The genomic region of *dmfrn *was subcloned in several steps to generate a genomic construct, tagged by a C-terminal fusion with the coding sequence of the fluorescent protein venus. For all PCR reactions, Phusion high fidelity polymerase (Finnzymes) was used with either *w^1118 ^*genomic DNA, or plasmid pHWV (Carnegie *Drosophila *Gateway^® ^Collection) as a template for *dmfrn *or *venus *respectively. Restriction digestions were carried out with enzymes from New England Biolabs. Arctic shrimp alkaline phosphatase and the Rapid DNA Ligation Kit were obtained from ROCHE Applied Science.

Using primers 5**' ACT AGT **CTA GGA GCA GCA GGC CCA C 3' (introducing *SpeI *and a stop codon in the first exon of *Gp93*) and 5' AAA A**AT CGA T**AA AA**G CTA GC**C GTG CTG AAG CCC CGC TCG 3' (introducing *NheI *and *ClaI*) the region from the first exon of *GP93 *to the end of the coding sequence of *dmfrn*, omitting the stop codon, was subcloned into pCR-XL-TOPO (Invitrogen) by TOPO cloning (Invitrogen). Using primers 5' AAA A**GC TAG C**AT GGT GAG CAA GGG CGA G 3' (introducing a *NheI *site) and 5' AAA A**AT CGA T**TC ACG TGG ACC GGT GCT T 3' (introducing a *ClaI *site), the coding sequence of the fluorescent protein venus was PCR amplified, and cloned in frame behind the coding sequence of dmfrn using restriction sites *NheI *and *ClaI*. Next, the 3'UTR of *dmfrn *was PCR amplified, using primers 5' AAA A**AT CGA T**AC GTA GGC GTC GCC GGT GG 3' (introducing *ClaI*) and 5' AAA A**GG TAC C**CG GAA ACA ATA AAA GGC AAT TGT TG 3' (introducing a *KpnI *site) and was cloned behind the coding sequence of *venus*, using the introduced *ClaI *site of the previous step and a *KpnI *site within the plasmid. Cloned fragments were sequenced after each step. Finally, the genomic construct was cloned into *pCasper4 *[52] using restriction sites *SpeI *and *KpnI *and the resulting plasmid was sent for co-injection with plasmid pΔ2-3 into *w^1118 ^*embryos for generation of transgenic flies at the Department of Developmental Biology, Wenner-Gren Institute, Stockholm University. Transgenic *dmfrn^venus ^*flies were verified by PCR and outcrossed for four generations to *w^1118 ^*flies.

### RT-RT PCR

RNA was extracted from tissues prepared from 2 to 3 days old virgin male *w^1118 ^*adult flies, raised at room temperature on potato food. Dissections were carried out in PBS buffer on a wax plate on ice and tissues were dissolved directly in 400 μL 1% 2-mercaptoethanol RLT buffer from the RNeasy Kit (QIAGEN). Heads and thoraxes were ripped open before lysis and disrupted using a micro pestle. Tissues from 20 flies were pooled per experiment and after passing lysates through QIAShredder columns (QIAGEN), RNA was purified from 350 μL flow through using the RNeasy Kit (QIAGEN). Integrity of RNA was analyzed spectrophotometrically and by agarose gelectrophoresis. cDNA was synthesized from 350 ng total RNA using the QuantiTect reverse transcription kit (QIAGEN) performing the gDNA wipeout reaction to remove genomic DNA contaminations. The cDNA synthesis reaction was also performed without QScript RTase and used as a negative control PCR reaction to test for gDNA contamination. The QuantiTect SYBR green kit (QIAGEN) was used for RT-RT PCR in a RotorGene 3000 (Corbett Research) thermocycler using primers for cDNAs of genes *Rp49 *[53], *dmfrn*, *Fer1HCH*, *Fer2LCH *[13], *Fer3HCH *(CG4349), forward 5'- GAA GGC ATC CCA CCA GTA TC-3', reverse 5'- GGC TGT GGT ACA CTG CTC AA-3' and *frataxin homolog *(CG8971) forward 5'- ACA AGC ACA GTG GTC AGT CG-3', reverse 5'- TAC AGT AGG GCA GGC GTA GG -3'.

### X-gal Staining

Testes were fixed in 3.7% (w/v) formaldehyde in PBS for 15-30 min at RT. Fixed testes were washed twice for 10 min with PBS, permeabilized for 20 min with PBST (PBS, 0.3% (v/v) Triton X-100) and then stained with staining solution (10% (w/v) 5-Bromo-4-chloro-3-indolyl β-D-galactoside (X-gal) in dimethyl sulfoxide was added to a final concentration of 0.2% (w/v) to preheated staining buffer (10 mM phosphate buffer pH 7.2, 150 mM NaCl, 1 mM MgCl_2_, 3 mM K_4_(Fe(II)(CN)_6_, K_3_(Fe(III)(CN)_6_, 0.3% (v/v) Triton X-100)) at 37°C until colorization was visible. Testes were first rinsed with NaCl-T (0.7% (w/v) NaCl, 0.3% (v/v) Triton X-100) then with water, mounted in PBS and imaged by conventional light microscopy.

### Testes preparation, testes squashes and fluorescence staining of testes and fluorescence microscopy

Testes of 1-3 days old virgin males were dissected out in a drop of PBS buffer using fine forceps (#5, FineScience Tools), transferred to a small drop of PBS on a microscope slide and carefully ripped open to release their content under the weight of a coverslip. Excess buffer was removed using a paper cloth, while observing the squash under phase contrast settings on a Leica TSC-SP microscope.

For fluorescence microscopy, testes were collected in Schneider Drosophila Medium (SDM) and stained with 100 nM MitoTracker Deep Red 633 nm (Invitrogen) in SDM for 2 hours. Testes were washed twice with PBS, fixed with 3.7% (w/v) formaldehyde in PBS, washed twice with PBS and permeabilized with 0.3% (v/v) Triton X-100 in PBS. After washing twice with PBS, testes were stained with Rhodamine Phalloidin (diluted 1:1000 in PBS; Invitrogen), washed three times with PBS and mounted in VECTASHIELD Mounting Medium with or without DAPI (Vector Labs). Specimens were either imaged using conventional fluorescent microscopy on a Leica TSC-SP or confocal laser scanning microscopy on a Leica TCSNT or a Zeiss LSM510 confocal microscope.

### Transmission electron microscopy

Glutharaldehyde fixed testes were dehydrated, embedded, sectioned and stained following standard procedures at the Biological Structure Analysis Facility, Uppsala University, Sweden. Sections were imaged using a Zeiss Supra35VP electron microscope.

## Authors' contributions

CM designed and performed all experiments, interpreted the results, drafted and wrote the manuscript. MIL discussed the experiments and results and critically read the manuscript. Both authors read and approved the final manuscript.

## Supplementary Material

Additional file 1 **Confirmation of fly lines used in the study, spermatogenesis defect of *dmfrn^venusb32^/+; Df(3R)ED6277 *flies and localization of ferritin during spermatogenesis.** Contains text, a table with primers and images. Click here for file
