# Supplementary material for: Drosophila mitoferrin is essential for male fertility: evidence for a role of mitochondrial iron metabolism during spermatogenesis
Source: BMC Dev Biol. 2010 Jun 21;10:68. doi: 10.1186/1471-213X-10-68 (PMC2905335; doi:10.1186/1471-213X-10-68)
Supplement: Additional file 1 — Confirmation of fly lines used in the study, spermatogenesis defect of dmfrnvenusb32/+; Df(3R)ED6277 flies and localization of ferritin during spermatogenesis. Contains text, a table with primers and images. [file 1471-213X-10-68-S1.PDF]

Additional methods

Genotyping

Genomic DNA (gDNA) was extracted by homogenizing 1 to 20 flies in 200 µL Buffer A (100 mM Tris-HCl, pH 7.5, 100 mM EDTA, 100 mM NaCl, 0.5% SDS). The homogenate was incubated at 65°C for 30 min. 400 µL Li/Ac solution (1 part 5 M potassium acetate freshly mixed with 2.5 parts 6 M LiCl) were added and incubated for >10 min on ice. Crude particles were sedimented at 16 000 x g for 15 min at room temperature in a table top centrifuge. 500 µL supernatant were transferred to a new tube and mixed with 300 µL isopropanol and centrifuged at 16 000 x g for 15 min at RT. The gDNA pellet was washed once in 800 µL 70% ethanol, dried and solubilized in 50 µL TE buffer (10 mM Tris-HCl and 1 mM EDTA, pH 8.0).

50-250 ng gDNA were used in PCR reactions with combinations of primers (Additional file1, table I) indicated in the text. PCR for one and two step confirmation of *Df(3R)ED6277* was carried out with Phusion high fidelity polymerase (Finnzyme). All other reactions were carried out with HotMasterTaq polymerase (5 PRIME).

Additional data

Additional table I: Primers used for genotyping. (see Additonal file 1, Figure A1 for primer positions).

Primer name	Primer sequence (5´ to 3´)
Ploc1F	GCACGAGGCTTGTTTATTGG
Ploc1R	AGTCCAGCGGATACATGACC
Ploc2F	AAGGCCACGTCACGAATAAC
InvRep	GACCACCTTATGTTATTTCATCATG
lacZori	GTTTTCCCAGTCACGACGTT
sh115LR-R	ATCTCGTAGGCGGCAAAGTA
6277.A	GTTGTTGGTGTCCCTCGG
W11678U	TCATCGCAGATCAGAAGCGG
W7500D	GTCCGCCTTCAGTTGCACTT
6277.B	GGTCGATGGTCTCCGTTG

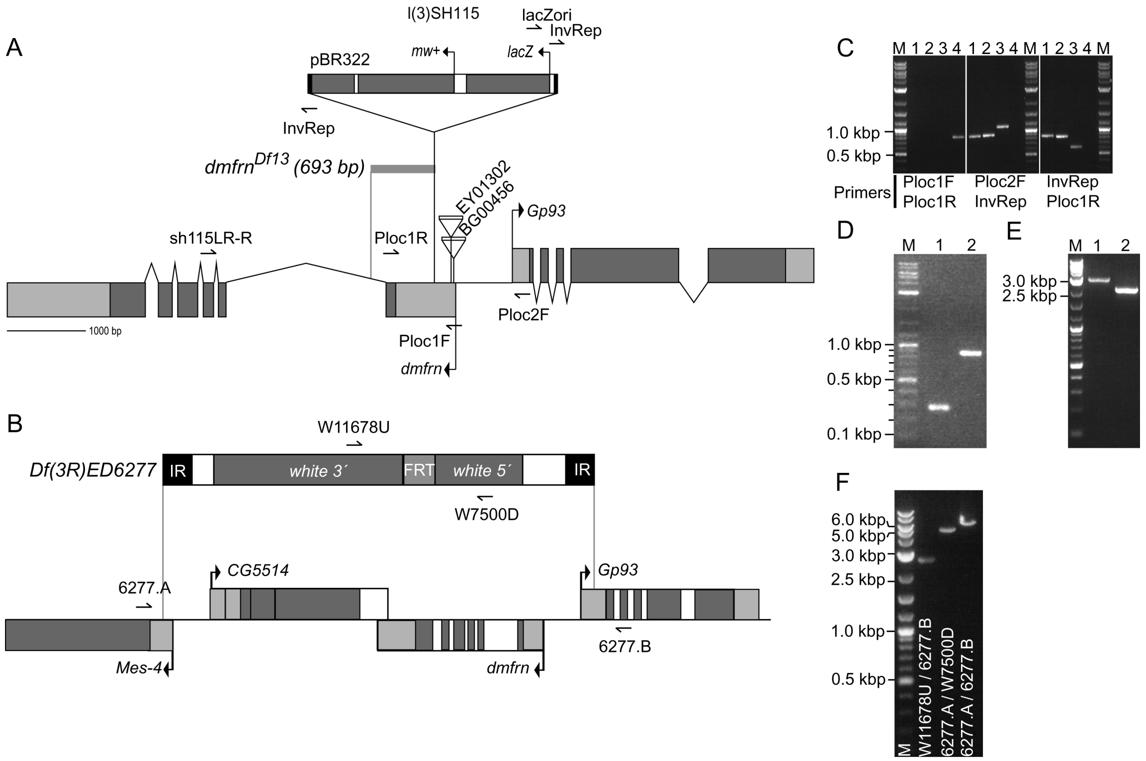

Additional Figure A1: A and **B:** Maps of *mitoferrin* gene region indicating positions of primers (half arrows with primer name, for primer sequences see Additional file 1 Table I) used to genotype fly strains. P-element insertion sites (**A**) in *Drosophila mitoferrin* and breakpoints of deletions *dmfrn*^{Df13} (**A**) and *Df(3R)ED6277*(**B**). **C:** Genotyping of *dmfrn*^{BG00456} (1), *dmfrn*^{EY01302} (2), *dmfrn*^{SH115} (3) and WT (4) using the primers indicated below the gel image for genotyping. **D:** Confirmation of the orientation of *P{lacW}mfrn*^{SH115} using primers lacZori / Ploc1F (1) and lacZori / Ploc2F (2). **E:** Comparison of PCR products from gDNA of *w*^{1118} (1) and *w*^{1118}; *dmfrn*^{Df13} (2) flies using primers Ploc1F and sh115LR-R. **F:** PCR confirmation of the deficiency *Df(3R)ED6277* by two step (primers W11678U x 6277B and 6277.A x W7500D) and one step (primers 6277.A x 6277.B) PCR.

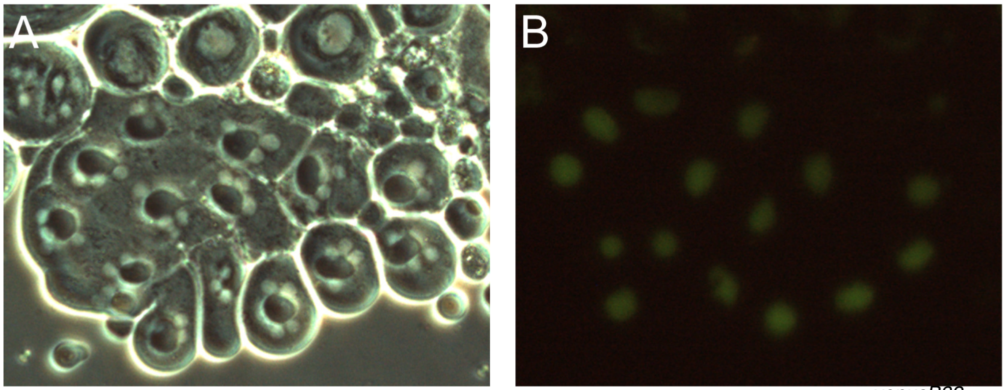

Additional Figure A2: Onion stage spermatids of $dmfrn^{venusB32}$/+; *Df(3R)ED6277* with giant mitochondrial derivative that associates with several nuclei of normal size. Phase contrast (A) and fluorescence (B) microscopy.

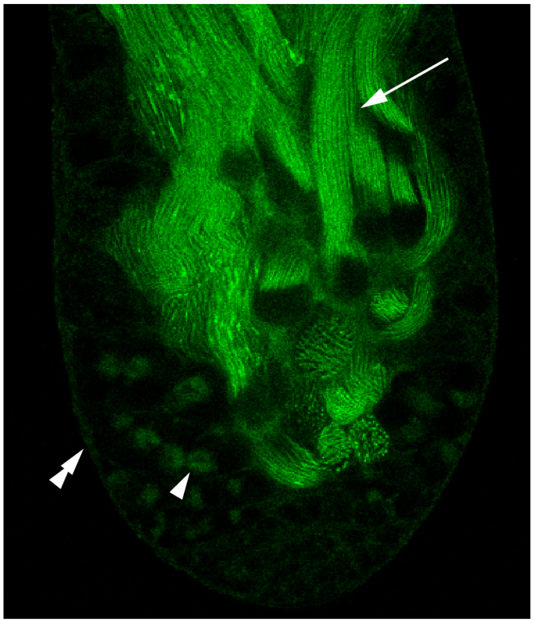

Additional Figure A3: Confocal laser scanning microspcopy of the tip of a testis of a $dmfrn^{venus}$ fly. The signal of dmfrn-venus protein is strongest in spermatids (arrow), but a clearly weaker signal can be detected in spermatocytes (single arrow head) and in the testis sheath (double arrow head) as well.

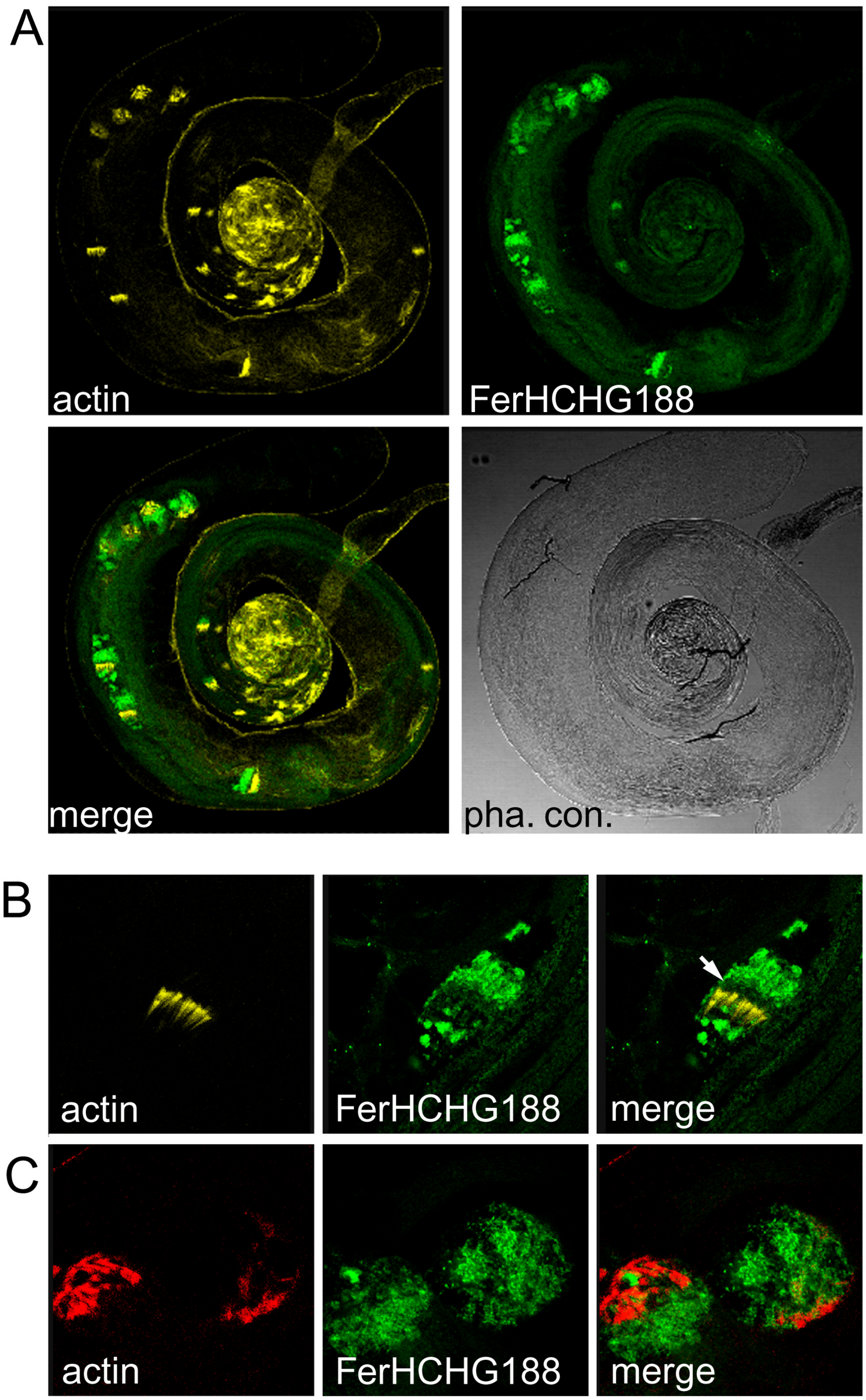

Additional Figure A4: Localization of FerHCH^{G188} protein in developing spermatids by laser scanning confocal microscopy. Overview of *FerHCH^{G188}/TM6c* testis (A), individualization complex (B) and formation of waste bag (C). FerHCH^{G188} accumulates in proximity to the actine cones of the individualization complex, in front of the mitochondrial

whorls. This is in agreement with the localization of *Drosophila* ferritin to the endoplasmatic reticulum and the localization of the endoplasmatic reticulum during spermatogenesis [1].

References

1. Dorogova, N., Nerusheva, O., Omelyanchuk, L.: **Structural organization and dynamics of the endoplasmic reticulum during spermatogenesis of *Drosophila melanogaster*: Studies using PDI-GFP chimera protein.** *Biochemistry (Moscow) Supplemental Series A: Membrane and Cell Biology* 2009, **3:**55-61.
